# Dietary replacement of peanut vines with fermented rice straw alters growth performance, nutrient digestibility, rumen fermentation characteristics in Hu lambs

**DOI:** 10.3389/fvets.2025.1720037

**Published:** 2025-12-16

**Authors:** Jing Wang, Han Zhang, Lu Li, Yufeng Li, Shengyong Mao, Yuyang Yin

**Affiliations:** 1 Huzhou Academy of Agricultural Sciences, Huzhou Municipal Bureau of Agriculture and Rural Affairs, Huzhou, China; 2 Laboratory of Gastrointestinal Microbiology, National Center for International Research on Animal Gut Nutrition, Nanjing Agricultural University, Nanjing, China; 3 Ruminant Nutrition and Feed Engineering Technology Research Center, College of Animal Science and Technology, Nanjing Agricultural University, Nanjing, China

**Keywords:** fermented rice straw, peanut vine replacement, fattening Hu lambs, rumen fermentation, rumen microbiota

## Abstract

Efficient utilization of agricultural by-products as alternative roughage sources is essential for sustainable ruminant production where high-quality forages are limited. This study evaluated the effects of replacing peanut vines with fermented rice straw on growth performance, nutrient digestibility, rumen fermentation, and microbial communities in growing Hu lambs. Thirty male Hu lambs (19.46 ± 1.15 kg) were randomly assigned to three groups for 56 days: CON (basal diet contained 10% peanut vines), ST50 (50% replacement of peanut vines with fermented rice straw), and ST100 (100% replacement). Compared with CON, final body weight and dry matter intake were not affected in ST50 and ST100 (*p* > 0.05), whereas feed conversion ratio increased in ST100 (*p* = 0.034). Apparent digestibility of DM, OM, CP, NDF, and ADF significantly declined in ST100 but remained unchanged in ST50 (*p* < 0.001). Rumen pH and NH3–N remained stable, while ST50 increased propionate (*p* = 0.008) and reduced valerate and the acetate-to-propionate ratio (*p* < 0.001 and *p* = 0.007). Total VFA concentration was lower in ST100 than in ST50. Rumen papillae density and surface area decreased in ST100 (*p* > 0.05). Sequencing of 16S rRNA indicated unchanged alpha diversity but modest separation in beta diversity, with core genera (e.g., *Prevotella, Ruminococcus, Succiniclasticum*) conserved. Indicator species analysis showed enrichment of *Selenomonas* and *Succinivibrionaceae_UCG-002* in CON, whereas ST100 was characterized by fiber-associated lineages, including *Anaerolineae, Family_XIII_AD3011_group*, and *Prevotellaceae_UCG-001/003*. Correlation network analysis revealed two opposing microbial clusters: one linked to propionate and NH_3_-N, and the other associated with acetate, butyrate, and TVFA. In conclusion, partial (50%) replacement maintained performance and improved rumen fermentation, whereas full replacement impaired digestibility. Future work should optimize fermentation techniques and microbial strategies to enhance fiber utilization.

## 1 Introduction

Sustained shortages of high-quality forages and fluctuation in feed prices limit both the profitability and resilience of the small ruminant industry. Peanut vines, a by-product of peanut harvesting, are widely used as unconventional roughage because of their relatively high crude protein content (1), good palatability, and moderate fiber levels (2–4). However, the supply and price of peanut vines vary considerably depending on the origin, collection and storage conditions, and competing uses, leading to uncertainty into year-round ration formulation in commercial farming systems. In contrast, rice straw is an abundant and inexpensive by-product in rice-growing regions, but its direct use in finishing lamb diets is limited by high lignification, recalcitrant cell walls, low crude protein, and poor palatability (5–7). It remains underutilized in the livestock industry. The degradability of rice straw is strongly constrained by its molecular architecture, particularly the ratio of cellulose, hemicellulose, and lignin. Variations in rice plant genetics can substantially alter lignocellulosic composition, which in turn affects structural integrity, enzymatic accessibility, and microbial degradability (8). Microbial fermentation has therefore emerged as a practical strategy to upgrade low-quality roughages by partially disrupting lignocellulosic structures (9), increasing soluble carbohydrates and degradable nitrogen, and enhancing aroma and palatability (10–12). Reported responses to fermentation include improved degradability of neutral detergent fiber (NDF) and acid detergent fiber (ADF), increased apparent crude protein, and shifts in rumen fermentation toward more efficient volatile fatty acid (VFA) profiles, with potential benefits for average daily gain (ADG) and feed efficiency (13).

Rumen fermentation efficiency depends on highly diverse and dynamic microbial community that regulate fiber degradation, nitrogen recycling, and VFA production. Spatial and temporal variations in these microbiota strongly influence feed adaptation and host nutrient utilization (14). Recent evidence also indicates that nutrient-specific factors—such as lipids and bioactive compounds—can modulate microbial metabolism and community structure, thereby altering fermentation pathways (15). These complex host–microbe–diet interactions highlight the complexity of predicting ruminant responses to new feed resources such as fermented rice straw.

Furthermore, rumen microbial activity contributes to systemic metabolic regulation through microbiota–metabolite crosstalk (16), in which short-chain fatty acids play a central role. In particular, butyrate functions not only as a key energy substrate for rumen epithelial cells but also as a regulator of epithelial integrity and barrier function (17). Therefore, investigating how microbiota and fermentation profiles respond to fermented rice straw replacement will help elucidate nutritional and physiological mechanisms behind feed efficiency and tissue health.

However, there remains limited consensus on optimal inclusion levels, dose–response relationships, and physiological threshold for incorporating fermented rice straw into finishing lamb diets under realistic replacement scenarios (9). If fermented rice straw can fully replace peanut vines without compromising growth performance and ideally while reducing ration costs, it would represent a more scalable and sustainable roughage option for the sheep industry (18).

Building on a validated preparation process for fermented rice straw, we adopted a replacement design in which fermented rice straw substituted peanut vines at two inclusion levels in finishing lamb diets. We hypothesized that, within a practical range, replacement would maintain or improve growth performance, enhance apparent total-tract nutrient digestibility, optimize rumen fermentation, and preserve metabolic health, while achieving better economic returns. Therefore, the present study aimed to determine an appropriate inclusion range and to characterize plausible mechanisms underlying performance responses, thereby providing evidence-based guidance for the commercial application of fermented rice straw.

## 2 Materials and methods

### 2.1 Animals, diets, and experimental design

The experimental protocol was approved by the Animal Care and Use Committee of Nanjing Agricultural University (protocol number: SYXK2017-0007).

A total of thirty healthy male Hu lambs (3 months of age, 19.46 ± 1.20 kg) in the fattening stage were used in this study. The animals were randomly assigned to three treatment groups, with 10 lambs per group. The experimental groups included a control group (CON, basal diet contained 10% peanut vines), 5% rice straw group (ST50, 50% replacement of peanut vines with fermented rice straw), and 10% rice straw group (ST100, 100% replacement). Each lambs was housed in an individual pen (1.5 × 2 m) with wooden slatted floors and had free access to drinking water. After the adaptation period (7 days), the experimental period spanned 56 days. All the lambs were fed twice daily at 07:00 and 16:00, ensuring a surplus of 5%−10%. All raw materials were procured from Da Bei Nong Technology Co., Ltd. in Anhui Province. Ingredients and chemical composition of the experimental diets are presented in Table 1. All lambs were uniformly dewormed prior to the experiment. During the feeding period, one Hu lamb in the ST100 group died accidentally due to causes unrelated to the experimental treatments. Therefore, subsequent analyses for this group were conducted with nine animals, whereas the CON and ST50 groups each included 10 animals. The difference in sample size was taken into account in the statistical analyses.

**Table 1:** Ingredients and chemical composition of the experimental diets.

**Items**	**Groups** ^*****a*****^		
	**CON**	**ST50**	**ST100**
**Ingredient, % of DM**		
Corn silage	25.00	25.00	25.00
Fermented rice straw	0.00	5.00	10.00
Rice straw	10.00	5.00	0.00
Corn meal	37.00	37.00	37.00
DDGS	6.00	6.00	6.00
Soybean meal	9.00	9.00	9.00
Rapeseed meal	4.00	4.00	4.00
Soybean hull	4.25	4.25	4.25
Urea	0.75	0.86	0.97
Rice husk powder	0.60	0.39	0.15
Calcium carbonate	1.15	1.25	1.38
Salt	0.50	0.50	0.50
Calcium monophosphate	0.75	0.75	0.75
Sodium bicarbonate	0.75	0.75	0.75
Premix^b^	0.25	0.25	0.25
Total	100.00	100.00	100.00
**Chemical composition, % DM**		
DM	73.50	70.81	68.13
CP	15.51	15.61	15.47
NDF	33.13	33.34	33.88
ADF	19.79	18.85	18.17
EE	3.53	3.26	2.80
Ash	6.96	7.82	8.38
ME (MJ/kg DM)	11.33	11.21	11.08

^a^CON (basal diet contained 10% peanut vines); ST50 (50% replacement of peanut vines with fermented rice straw); ST100 (100% replacement). ^b^Premix contained the following ingredients: vitamin A, 150,000 KIU/kg; vitamin D, 100,000 KIU/kg; vitamin E, 600 IU/kg; Mn, 1.8 g/kg; Cu, 0.3 g/kg; Co, 15 mg/kg; I, 25 mg/kg; Se, 10 mg/kg; Fe, 1.2 g/kg. An additional row for dry matter (DM) has been added to the chemical composition section of Table 1: CON = 73.50%, ST50 = 70.81%, and ST100 = 68.13%.

### 2.2 Sampling and measurement

#### 2.2.1 Growth performance

On the first day prior to the formal experimental period and on the last day of the experimental period, the lambs were weighed before the morning feeding to calculate the average daily gain (ADG). ADG for each lamb was calculated as (final body weight—initial body weight)/feeding days. The mean ADG of each treatment group was obtained from individual ADG values, with individual animals considered as experimental units for statistical analysis. Additionally, the diet offered and the orts were measured daily for each group of lambs to assess dry matter intake (DMI) and the gain -to- feed ratio (G /F) of the Hu lambs.

#### 2.2.2 Apparent nutrient digestibility

During the digestibility assessment, fecal samples were collected from each lambs using the spot sampling method for three consecutive days after the feeding period. Approximately 100 g of fresh feces were obtained at each sampling (morning and evening). All subsamples from the same animal were thoroughly mixed to form a “composite sample.” The samples were thoroughly homogenized, and a portion was combined with an equal volume of 10% dilute sulfuric acid for nitrogen fixation. All fecal samples were stored at −20 °C.

During the concluding 3 days of the experiment, feed samples were collected, thoroughly mixed, and subjected to the quartering method for sampling. After the experiment, both fecal and feed samples were dried in an oven at 65 °C for 48 h. Subsequently, the samples were ground using a Cyclotec mill (Tecator 1,093; Tecator AB, Höganäs, Sweden) with a 40-mesh sieve for conventional nutrient analysis. A portion of the air-dried feed was further dried at 105 °C for 3 h to determine the dry matter content. The methods used to measure neutral detergent fiber (NDF) and acid detergent fiber (ADF) were based on Van Soest et al. (19), while the crude protein (CP), crude fat (EE), and ash content in feed and feces were measured according to AOAC (20). The apparent digestibility was calculated using acid-insoluble ash (AIA) as a marker, following the methods of Van Keulen and Young (21). The calculation formula is as follows:


$$\begin{array}{l} \text{Nutrient digestibility }\left(\%\right)=\;\\ \;\left[\begin{array}{c} 1-\left(\frac{\text{AIA concentration in feed}}{\text{AIA concentration in feces}}\right)\times \\ \left(\frac{\text{Nutrient concentration in feces}}{\text{Nutrient concentration in feed}}\right) \end{array}\right]\times 100\% \end{array}$$

#### 2.2.3 Collection, fixation, and blinded measurement of rumen papillae

From the dorsal sac of each lambs' rumen, a ~1 × 1 cm epithelial specimen was collected, and the total number of papillae within this area was recorded as *n*. From each specimen, three papillae were randomly selected at each of five regions (the four corners and the center), and their length and width were measured with a calibrated vernier caliper (0–150 mm, resolution 0.01 mm, accuracy ±0.02 mm; Manufacturer, Model, Country); damaged or fused papillae were excluded and replaced at random within the same region. The absorptive surface area of an individual papilla was approximated using a two-sided rectangular model as *S*_*i*_ = length_*i*_ × width_*i*_ × 2. The mean papillary surface area for the specimen was calculated from the 15 papillae (*S*_mean_), and the absorptive surface area per unit epithelial area (per cm^2^) was computed as *S*_unit_ = *S*_mean_ × *n*. All measurements were performed by the same trained operator to minimize variability, and results were analyzed and reported with the animal as the experimental unit.

#### 2.2.4 Rumen fermentation parameters

On the last day of the formal experimental period, rumen fluid was collected 2.5 h post-morning feeding using an oral stomach tube. The first 50 ml of the sample was discarded to minimize contamination from saliva. Following immediate filtration through four layers of cheesecloth, the pH of the rumen fluid was measured using a portable pH meter (HI-9024C, HANNA Instruments, United States). The remaining samples were subpackaged and stored at −20 °C for subsequent analysis. 1 ml of rumen fluid was mixed with 0.2 ml of 25% (w/v) orthophosphoric acid and analyzed for VFA concentrations using gas chromatography (GC-14B, Shimadzu, Japan) (22). The ammonia nitrogen (NH_3_-N) concentration in the rumen fluid was measured using a colorimetric method (23).

#### 2.2.5 DNA extraction, 16S rRNA amplicon sequencing, and bacterial composition analysis

Total microbial genomic DNA was extracted from rumen content samples using the E.Z.N.A.^^®^^ soil DNA Kit (Omega Bio-Tek, Norcross, GA, United States) according to the manufacturer's instructions. The quality and concentration of DNA were determined by 1.0% agarose gel electrophoresis and a NanoDrop^^®^^ ND-2000 spectrophotometer (Thermo Scientific Inc., United States) and kept at −80 °C prior to further use. The hypervariable region V3-V4 of the bacterial 16S rRNA gene was amplified with primer pairs 338F: ACTCCTACGGGAGGCAGCAG and 806R: GGACTACHVGGGTWTCTAAT (24) by an ABI Gene Amp^^®^^ 9,700 PCR thermocycler (ABI, CA, United States). Purified amplicons were pooled in equimolar amounts and paired-end sequenced on an Illumina Mi Seq PE300 platform/Nova Seq PE250 platform (Illumina, San Diego, USA) according to the standard protocols by Majorbio Bio-Pharm Technology Co. Ltd. (Shanghai, China).

Raw FASTQ files were de-multiplexed using an in-house Perl script, then quality-filtered by fastp version 0.19.6 (25) and merged by FLASH version 1.2.7 (26) with the following criteria: the 300 bp reads were truncated at any site receiving an average quality score of < 20 over a 50 bp sliding window, and the truncated reads shorter than 50 bp were discarded. Reads containing ambiguous characters were also discarded. Only overlapping sequences longer than 10 bp were assembled according to their overlapped sequence. The maximum mismatch ratio of the overlap region is 0.2. Reads that could not be assembled were discarded. Then the optimized sequences were clustered into operational taxonomic units (OTUs) using UPARSE 7.1 (27) with a 97% sequence similarity level. The most abundant sequence for each OTU was selected as a representative sequence. On the basis of the above analyses, a series of in-depth statistical and visual analyses, such as multivariate analysis and difference significance test, were conducted on the community composition of multiple samples. Alpha diversity was calculated using Qiime software (Version 1.9.1), and differences between groups were analyzed using R software (Version 2.15.3). Bray-Curtis distances were computed using the default script from the Phyloseq package to measure beta diversity. Principal Component Analysis (PCoA) was conducted using the ade4 and ggplot2 packages of R software.

### 2.3 Statistical analyses

The growth performance, apparent digestibility, and rumen fermentation parameters of Hu lambs were analyzed using one-way ANOVA in SPSS 26.0, and multiple comparison tests (SNK method) were performed. Covariance analysis was used for the FBW, with the covariate being the IBW. The differences in alpha diversity indicators and relative abundance of microbial communities were analyzed using non parametric tests (Kruskal Wallis). Spearman's rank correlation coefficients were calculated between the relative abundances of all pairs of genera using the Bioincloud (Gidio) online platform (https://www.bioincloud.tech). Only significant correlations (*p* < 0.05) with a Spearman's correlation coefficient |*R*| > 0.6 were retained for further analysis. The significance level *p* < 0.05 indicates significant differences, while *p* < 0.01 indicates extremely significant differences in the data.

## 3 Results

### 3.1 Growth performance

No significant differences in initial body weight, final body weight, ADG and DMI were observed among treatments (*p* > 0.05, Table 2). However, ST100 group showed a significant reduction in feed conversion ratio (G/F) compared with both the CON and ST50 groups, indicating lower feed efficiency at the higher supplementation level (*p* < 0.05).

**Table 2:** Effects of different inclusion levels of peanut vines on growth performance in fattening Hu lambs.

**Items**	**Groups**			**SEM**	***p*-Value**
	**CON**	**ST50**	**ST100**		
Initial BW, kg	19.19	19.41	19.80	0.213	0.538
Final BW, kg	34.43	35.46	35.17	0.493	0.706
DMI, g/days	1,233.75	1,346.79	1,396.71	30.651	0.096
ADG, g/days	272.14	286.61	274.40	7.353	0.715
G/F	0.220^*a*^	0.214^*a*^	0.196^*b*^	0.004	0.034

CON (basal diet contained 10% peanut vines); ST50 (50% replacement of peanut vines with fermented rice straw); ST100 (100% replacement). G/F, feed conversion ratio (gain-to-feed ratio). Values are expressed as mean; CON (n = 10), ST50 (n = 10), ST100 (n = 9). Values within the same row bearing different superscript letters (a, b) are significantly different (*p* < 0.05).

### 3.2 Apparent digestibility of nutrients

As shown in Table 3, nutrient intake showed increasing trends in DM, CP, and NDF with supplementation, whereas EE intake decreased. However, apparent digestibility of DM, OM, CP, NDF, and ADF was significantly reduced in the ST100 group compared with CON and ST50 (*p* < 0.01). EE digestibility also tended to decrease (*p* = 0.053).

**Table 3:** Effects of different inclusion levels of peanut vines on nutrient apparent digestibility in fattening Hu lambs.

**Items**	**Groups**			**SEM**	***p*-Value**
	**CON**	**ST50**	**ST100**		
**Organic nutrients digestibility, %**					
DM	80.96^a^	80.43^a^	76.37^b^	0.422	0.001
OM	83.93^a^	83.00^a^	79.60^b^	0.455	0.005
CP	79.84^a^	79.88^a^	74.56^b^	0.739	0.002
NDF	65.00^a^	64.35^a^	58.87^b^	1.326	0.002
ADF	70.09^a^	68.39^a^	62.93^b^	1.325	0.006
EE	86.62	85.80	82.10	1.317	0.053

CON (basal diet contained 10% peanut vines); ST50 (50% replacement of peanut vines with fermented rice straw); ST100 (100% replacement). DM, dry matter; OM, organic matter; CP, crude protein; NDF, neutral detergent fiber; ADF, acid detergent fiber; EE, ether extract. Values are expressed as mean; CON (n = 10), ST50 (n = 10), ST100 (n = 9). Values within the same row bearing different superscript letters (a, b) are significantly different (*p* < 0.05).

### 3.3 Rumen-reticulum histomorphometry

Replacing peanut vines with rice straw reduced the papillae density in the rumen significantly (*p* < 0.01, Table 4) but had no significant effect on papilla height and thickness. In addition, the rumen surface area of the three groups showed a decreasing trend (*p* = 0.085).

**Table 4:** Effects of different inclusion levels of peanut vines on rumen-reticulum morphology in fattening Hu lambs.

**Items**	**Groups**			**SEM**	***p*-Value**
	**CON**	**ST50**	**ST100**		
Papillae density (*n*/cm^2^)	62.71^a^	46.50^b^	46.50^b^	2.486	0.008
Papillae height (mm)	4.35	3.81	3.47	0.245	0.412
Papillae width (mm)	1.96	2.04	1.95	0.060	0.823
Surface area (mm^2^/cm^2^)	1,059.23	775.69	633.46	78.733	0.085

CON (basal diet contained 10% peanut vines); ST50 (50% replacement of peanut vines with fermented rice straw); ST100 (100% replacement). Values are expressed as mean; CON (n = 6), ST50 (n = 6), ST100 (n = 6). Values within the same row bearing different superscript letters (a, b) are significantly different (*p* < 0.05).

### 3.4 Rumen pH and fermentation parameters

**Figure 1:**
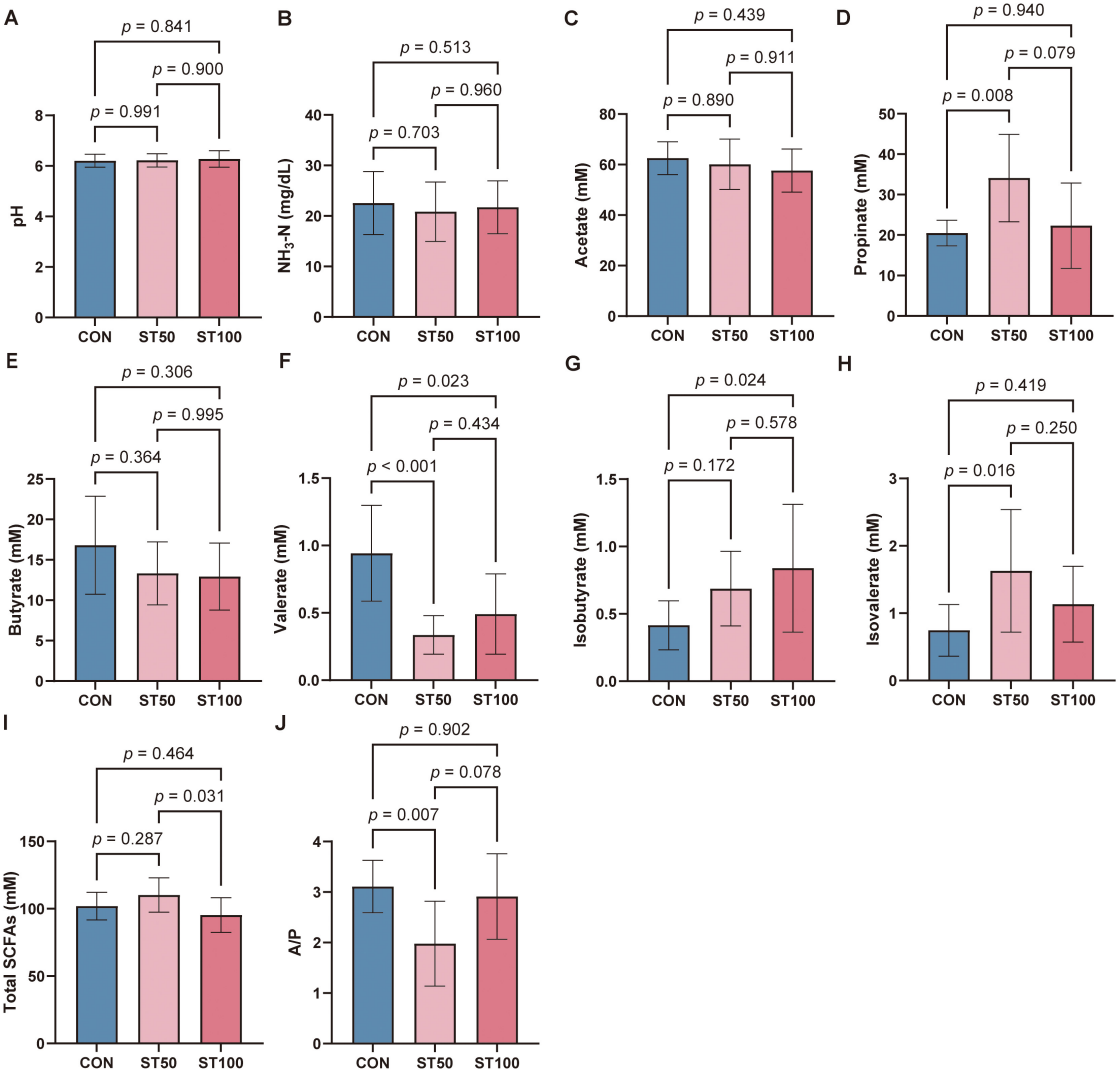
Rumen pH and fermentation parameters of Hu lambs fed fermented rice straw. **(A)** Rumen pH. **(B–I)** Rumen fermentation parameters. **(J)** A/P = acetate to propionate ratio. Data are expressed as the mean ± SD [CON and ST50 group (*n* = 10), ST100 group (*n* = 9)]. Conduct significance analysis of rumen pH and fermentation parameters using one-way ANOVA. *, *p* < 0.05; **, *p* < 0.01; ***, *p* < 0.001. SCFAs, short-chain fatty acids.

To further determine the influence of different inclusion levels of peanut vines on rumen fermentation, we analyzed parameters from ruminal fluids. The pH of ruminal fluids in the three groups was nearly identical (*p* > 0.05, Figure 1A). Moreover, the NH_3_-N concentrations tended to be lower in the ST50 and ST100 groups compared with the CON group, with no significant differences observed (Figure 1B). Following the replacement of peanut vines with fermented straw, the concentrations of acetate and butyrate in the rumen were lower than those in the CON group, although the differences were not statistically significant. In the ST50 group, however, propionate concentration was significantly higher, while valerate concentration and the acetate-to-propionate ratio (A/P) were significantly lower compared with the CON group (Figures 1C–F, J). Regarding branched-chain fatty acids, the ST100 group had higher levels of isobutyrate than the CON group (*p* < 0.05), and the ST50 group had higher levels of isovalerate than the CON group (*p* < 0.05). The remaining comparisons were not significant (Figures 1G, H). Furthermore, the total volatile fatty acids (TVFA) concentration in the ST100 group was significantly lower than that in the ST50 group, but did not differ significantly from the CON group (*p* < 0.05; Figure 1I).

### 3.5 Rumen microbial diversity

**Figure 2:**
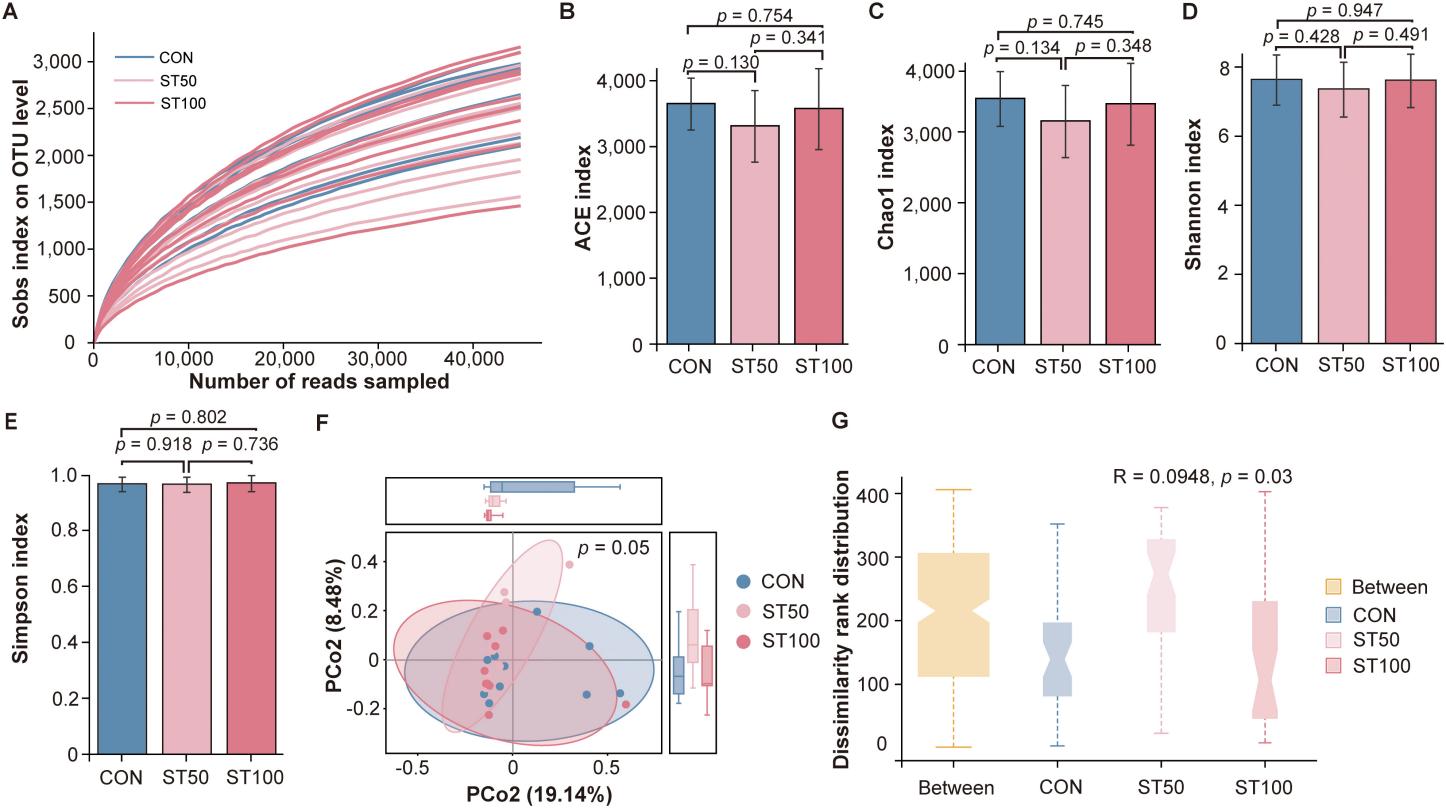
Effects of different inclusion levels of peanut vines on rumen microbial diversity in fattening Hu lambs. **(A)** Rarefaction curves of microbiome data measured by Sob index at the OTU level. **(B–E)** Alpha diversity analysis. **(F)** Principal coordinate analysis (PCoA) of the microbiome data. **(G)** Anosim analyze. The grouped “notched boxplots” display the median (at the notch), interquartile range (the box), and extremes (dashed whiskers). “Between” represents the overall distribution across all samples. Data are shown as individual raw values.

To determine whether replacing peanut vines with fermented rice straw affects the composition of the rumen microbiota, we performed 16S rRNA sequencing on rumen fluids from each group. The rarefaction curves (indicated by the Sobs index at the OTU level) of all samples exhibited a steady pattern when the number of reads sequenced exceeded 10,000 (Figure 2A), thus indicating the microbiome sequencing data were quantitatively adequate Subsequently, we assessed the richness and diversity of microbiota in the three groups. Across all three metrics, the distributions of values largely overlapped among groups, and no statistically significant differences were detected: ACE (Figure 2B), Chao1 (Figure 2C), Shannon (Figure 2D), and Simpson (Figure 2E). The above results hint that replacing peanut vines with fermented rice straw did not alter the richness and diversity of ruminal fluid microbiota in fattening Hu lambs. We also performed b-diversity analysis (i.e., principal coordinate analysis, PCoA) to evaluate the general similarity of microbiota among groups (Figure 2F). ANOSIM detected a small yet statistically significant group effect with *R* = 0.0948 and *p* = 0.03 (Figure 2G), suggesting that although between-group compositional differences are modest in magnitude, there is a detectable deviation from complete similarity at the community level.

### 3.6 Composition of the microbial communities is affected by the different inclusion levels of peanut vines in fattening Hu lambs

**Figure 3:**
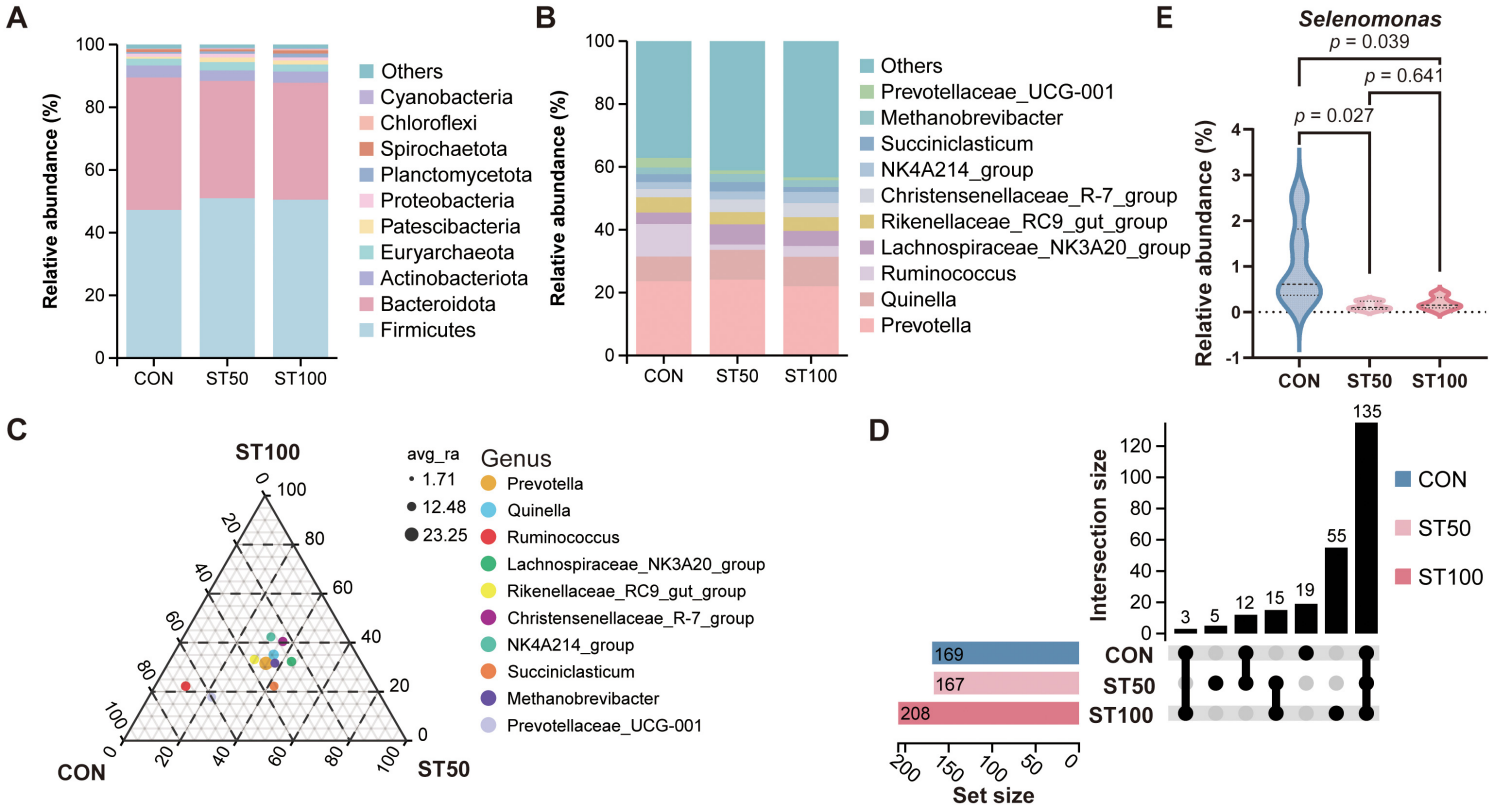
Effects of peanut vines on the relative abundance (relative abundance >1% in at least one treatment) of rumen bacteria at **(A)** phylum and **(B)** genus levels in fattening Hu lambs. **(C)** Ternary plot showing the relative abundance distribution of major bacterial genera among three groups. Each point represents a genus, with point size indicating average relative abundance (avg_ra), and colors distinguishing different genera. **(D)** An upset plot showed the number of shared and unique species in each experimental group. **(E)** Differential rumen bacteria at the genus level.

To clarify whether replacing peanut vines with fermented rice straw alters the rumen microbial composition, we analyzed the microbial communities of the three groups. At the phylum level, 10 phyla had a relative abundance >1%. The relative abundance of *Firmicutes, Bacteroidetes* and *Actinobacteriota* was high, but there were no significant changes in the microorganisms in the ST50 and ST100 groups compared to CON group (*p* > 0.05; Figure 3A). Subsequently, we characterized the microbial composition of ruminal fluids collected from post-weaned Hu lambs at the genus level. The top ranked microbes (relative abundances > 1%) include: *Prevotella, Quinella, Ruminococcus, Christensenellaceae_R-7_group, Succiniclasticum, Lachnospiraceae_NK3A20_group* and *NK4A214_group* (Figure 3B). All three groups were dominated by a core community specialized in carbohydrate utilization: high-abundance *Prevotella* and *Quinella* fermented starch and soluble polysaccharides; *Ruminococcus* and *Christensenellaceae_R-7_group* participated in fiber degradation; *Succiniclasticum* together with the *Lachnospiraceae_NK3A20_group* and *NK4A214_group* facilitated the conversion of succinate into propionate and contributed to the production of other short-chain fatty acids such as butyrate. Overall, the three groups shared these major genera, indicating the presence of a stable core microbiota. Ternary plot analysis revealed that the dominant genera were primarily distributed near the center of the triangle, indicating relatively balanced abundances across groups (Figure 3C). Among them, *Prevotella* and *Ruminococcus* represented the major genera with consistently high abundances. In contrast, *Quinella* was more enriched in the CON group, while *Christensenellaceae_R-7_group* and *NK4A214_group* tended to cluster in the ST50 and ST100 groups. Several low-abundance genera, such as *Succiniclasticum* and *Prevotellaceae_UCG-001*, showed group-specific shifts, suggesting that although the overall community structure remained relatively stable, certain taxa responded sensitively to the treatments. In the genus-level shared–unique analysis (Figure 3D), the numbers of detected genera were 169 in CON group, 167 in ST50 group, and 208 in ST100 group, with the largest intersection comprising ~135 genera common to all three groups, while genera unique to a single group or shared by only two groups were comparatively few, indicating a predominantly shared core microbiota and suggesting that the treatments primarily modulated the relative abundances of core taxa rather than introducing distinct group-specific genera. *Selenomonas*, a key lactate-utilizing, propionate-producing genus in the rumen, showed significantly lower relative abundance in ST50 group and ST100 group than in CON group, implying reduced lactate clearance and propionate formation under the straw-fermentation replacement diets (Figure 3E).

### 3.7 Relationships between major rumen bacteria and fermentation parameters

**Figure 4:**
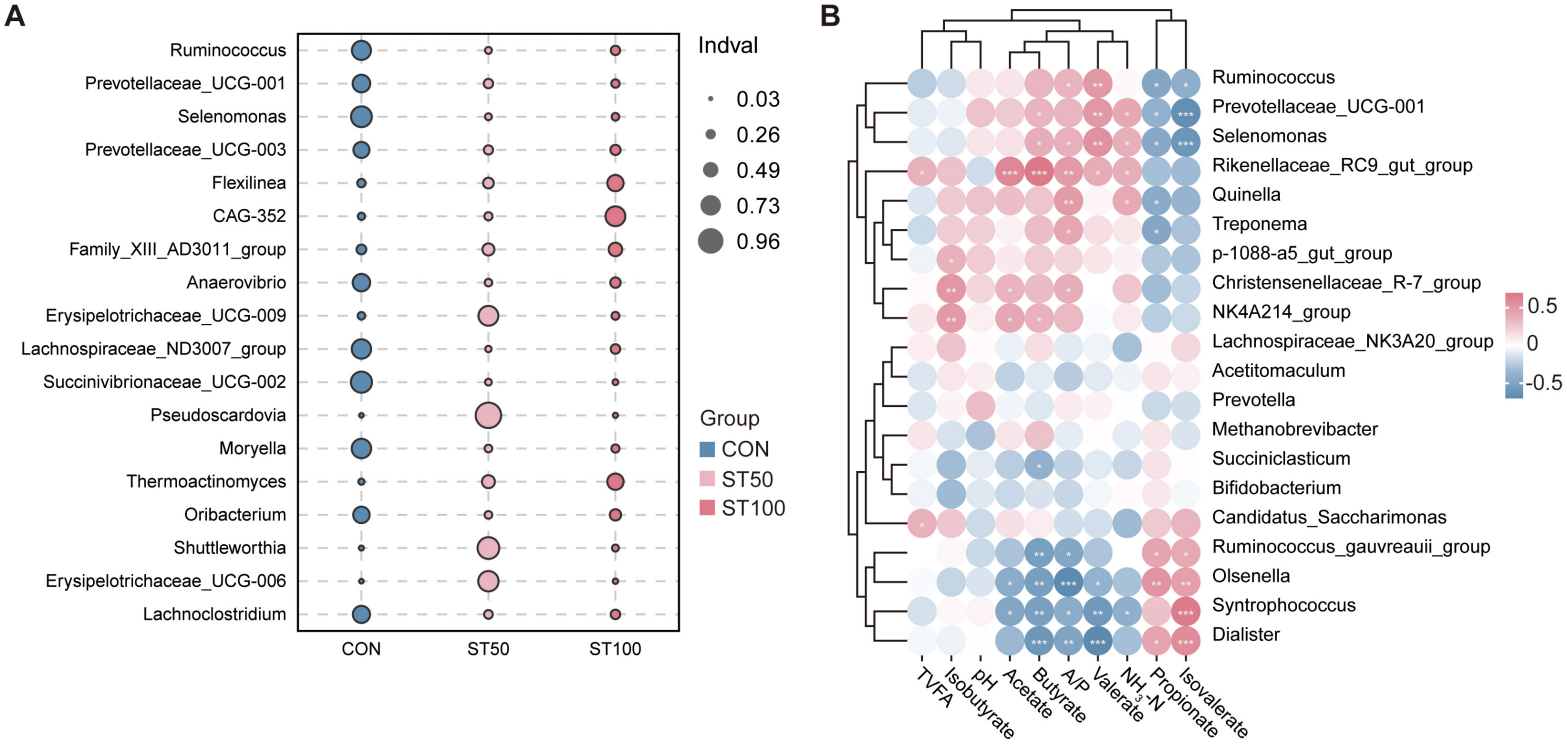
Relationships of bacterial communities and VFA fermentation parameters. **(A)** Indicator species analysis identified bacterial genera significantly associated with each group; bubble color denotes group, bubble size is proportional to Indval (legend at right), and only taxa significant by permutation (999) with BH correction are shown (*q* < 0.05). **(B)** The heatmap shows that the correlation between the predominant rumen bacteria (relative abundance of the top 20) and VFA fermentation parameters. *p < 0.05; **p < 0.01; ***p < 0.001.

Indicator species analysis at the genus level differentiated the three diets. Compared with CON group, seven genera were indicative of the control diet, including *Selenomonas, Succinivibrionaceae_UCG-002, Ruminococcus, Moryella, Lachnospiraceae_ND3007_group, Anaerovibrio*, and *Lachnoclostridium* (all *p* ≤ 0.023). ST50 group was characterized by *Erysipelotrichaceae_UCG-009, Shuttleworthia*, and *Pseudoscardovia* (*p* ≤ 0.012). ST100 group was associated with *Flexilinea, CAG-352*, F*amily_XIII_AD3011_group, Oribacterium, Thermoactinomyces, Prevotellaceae_UCG-003*, and P*revotellaceae_UCG-001* (*p* ≤ 0.023). Overall, lactate-utilizing taxa (e.g., *Selenomonas* and *Succinivibrionaceae_UCG-002*) typified CON group, whereas several fiber/complex carbohydrate–associated lineages typified ST100 group (Figure 4A).

Spearman correlation analysis was conducted to investigate the relationships between rumen VFA and the top 20 ranked bacterial genera based on relative abundance. The clustered correlogram shows a clear bifurcation of taxa with opposing association patterns along the VFA spectrum. One cluster comprising genera such as *Ruminococcus, Prevotellaceae_UCG-001, Selenomonas*, and members of the *Rikenellaceae_RC9_gut_group* exhibits predominantly positive correlations with propionate, isovalerate, and NH_3_-N, accompanied by negative correlations with pH and major SCFAs (notably acetate and butyrate). In contrast, a second cluster including *Bifidobacterium, Succiniclasticum, Methanobrevibacter, Dialister*, and related taxa displays positive associations with acetate, butyrate, TVFA, and pH, while showing inverse relationships with branched-chain VFAs (valerate/isovalerate) and nitrogenous indices. These findings suggest a potential role of rumen microorganisms in regulating rumen fermentation (Figure 4B).

## 4 Discussion

### 4.1 Growth performance

Final body weight and dry matter intake did not differ among groups, indicating that diet palatability and short-term intake regulation were not markedly influenced by the replacement of peanut vines with fermented rice straw. However, the ST100 group exhibited a lower feed conversion ratio (G/F) compared with the CON and ST50 groups, suggesting reduced feed efficiency at the higher supplementation level. This reduction in efficiency may be related to changes in rumen fermentation activity, nutrient digestibility, or microbial composition when a larger proportion of fermented rice straw was included in the diet.

### 4.2 Nutrient digestibility

This pattern was consistent with the digestibility data, where apparent digestibility of DM, OM, CP, NDF, and ADF declined significantly in ST100 group compared with CON group and ST50 group. Reduced digestibility under high straw inclusion is in line with the well-recognized limitations of crop residues, which are typically more lignified and contain higher silica levels than legume residues, thereby restricting microbial access to structural carbohydrates (28–30). Although fermentation pretreatment may partially improve straw quality (31, 32), the process used here did not sufficiently enhance fiber degradability at a 10% inclusion level to sustain nutrient capture.

In ruminants, modest inclusion of treated straw can maintain performance when the basal diet supplies adequate fermentable carbohydrates and nitrogen to support fibrolytic activity (33). Peanut vines, being protein-rich and highly degradable (7, 11, 34), likely supplied rapidly available nitrogen and soluble carbohydrates that facilitated rumen microbial activity. Their partial removal may therefore have reduced nitrogen availability, as suggested by the trend toward lower ruminal NH_3_–N concentrations in ST50 and ST100, which approached the critical threshold (5–8 mg/dl) required for optimal fiber digestion (35–37). This reduction in digestibility suggests a limitation in rumen microbial adaptation under full substitution. Future strategies may consider supplementing with natural plant-derived compounds to modulate microbial fermentative activity and redox balance, thereby improving nutrient utilization efficiency (38).

### 4.3 Rumen epithelial morphology

Rumen papillae adapt to fermentation intensity and butyrate exposure, both of which stimulate epithelial growth and ketogenesis (39, 40). In the present study, papillae density and surface area were reduced in ST100 compared with CON group, consistent with lower levels of TVFA and butyrate. Reduced fermentability likely limited epithelial stimulation and absorptive capacity, explaining the poorer feed conversion in ST100. Similar observations have been reported where low fermentable carbohydrate intake impaired papillary development independent of pH (41, 42). Comparable structural responses have also been documented in monogastric species, where enhanced epithelial morphology was observed under improved nutrient availability and fermentative balance (43). Although the physiological contexts differ, such findings provide useful insight into how epithelial tissues may adapt morphologically to dietary and microbial cues in ruminants. Future studies should explore the molecular and regenerative mechanisms underlying these interactions, as recent work has demonstrated that microbial signaling pathways can promote epithelial regeneration and resilience under dietary stress (44).

### 4.4 Fermentation profiles and metabolic implications

Despite no significant change in pH, the ST50 group diet increased propionate and reduced valerate and A/P. An increased propionate proportion generally reflects a shift toward succinate- and acrylate-pathway activity and can improve energetic efficiency under moderate concentrate supply (45). Lower A/P also implies reduced hydrogen availability for methanogenesis (45, 46). The reduction in valerate may reflect altered amino acid fermentation and more efficient nitrogen capture (47). Conversely, ST100 showed reduced TVFA, indicating limited fermentable substrate or microbial efficiency. Overall, moderate substitution redirected fermentation toward propionate, whereas excessive replacement constrained total fermentation.

### 4.5 Community structure: stability of the core and targeted shifts in low-abundance taxa

Alpha diversity did not differ among groups, and dominant genera—*Prevotella, Quinella, Ruminococcus, Christensenellaceae_R-7_group*, and *Succiniclasticum*—were shared and centrally positioned in the ternary plot, indicating a resilient core microbiota. This resilience is typical of the rumen, where functional redundancy and substrate cross-feeding buffer modest dietary changes (48, 49). Although β-diversity differed significantly, the effect size was small, suggesting limited reorganization mainly within subdominant taxa. These subtle yet structured shifts likely represent ecological fine-tuning to altered substrate supply. Differential responses of *Selenomonas* and *Prevotellaceae_UCG-001/003* exemplify how minor compositional shifts can underpin functional adaptation (50).

As *Selenomonas ruminantium* is a key lactate consumer and propionate producer (51, 52), its reduction suggests decreased availability of soluble carbohydrates with reduced peanut vines. Yet higher propionate in ST50 suggests compensation by other propionate producers (e.g., *Prevotellaceae_UCG-001/003*) or succinate-converting taxa such as *Succiniclasticum*. This increase in propionate proportion suggests a shift in hydrogen flow toward alternative reducing pathways, which could potentially decrease methanogenesis in the rumen by reducing hydrogen availability for methanogenic archaea. Interestingly, the correlation analysis grouped *Ruminococcus, Prevotellaceae_UCG-001*, and *Selenomonas* with propionate, isovalerate, and NH_3_-N and inversely with acetate/butyrate and pH. This pattern reflects their preference for readily fermentable carbohydrates and link to proteolysis/deamination pathways that generate branched-chain VFAs (53). *Succiniclasticum* converts succinate to propionate, yet its positive association with acetate and butyrate may arise from tight co-occurrence with fibrolytic and butyrogenic taxa and from system-level balancing where high TVFA states coincide with multi-product fermentation (54, 55). Overall, the weak β-diversity shift coupled with distinct taxonomic responses suggests that functional reorganization, rather than wholesale community change, underlies the observed fermentation patterns. Such functional reorganization may contribute not only to metabolic adaptation but also to subtle modulation of rumen epithelial morphology and absorptive capacity, as microbial regulation has been linked to reduced epithelial inflammation and improved papilla responsiveness under dietary interventions (56). Future inference based on tools such as PICRUSt2 or FAPROTAX could help verify these potential functional adjustments.

### 4.6 Indicator taxa and ecological interpretation

Indicator species analysis highlighted diet-specific microbial signatures. In CON group, enrichment of *Selenomonas* and *Anaerovibrio* aligns with higher availability of soluble sugars, fatty acids, and lactate management typical of legume-rich forages; *Anaerovibrio* hydrolyzes triacylglycerols and supports biohydrogenation (57). As shown in Figure 3E, the relative abundance of *Selenomonas* in the ST50 and ST100 groups was significantly reduced, which may weaken hydrogen uptake capacity and redox balance, thereby impairing fermentation stability and promoting the accumulation of intermediate acids under nutrient stress or low fermentability conditions. Such changes could partly explain the lower overall fermentative efficiency observed in high-replacement diets. ST50 group characterized by *Erysipelotrichaceae_UCG-009, Shuttleworthia*, and P*seudoscardovia*, consistent with adaptation to intermediate carbohydrate niches (58). In ST100 group, indicator lineages—*Flexilinea* (*Anaerolineae*), *CAG-352, Family_XIII_AD3011_group, Oribacterium, Thermoactinomyces, Prevotellaceae_UCG-003*, and *Prevotellaceae_UCG-001*—point toward complex polysaccharide deconstruction and possibly thermotolerant/firmly attached niches on fibrous particles. *Anaerolineae* have been repeatedly associated with lignocellulose degradation and biofilm formation in anaerobic digesters and the rumen (59). *Family_XIII* members and *Oribacterium* have been reported in forage-rich diets and may contribute to pectin and hemicellulose utilization (60, 61). The prevalence of these taxa in ST100 group supports a shift toward fiber-oriented fermentation, albeit with lower overall digestibility and epithelial stimulation, reflecting structural adaptation rather than efficient conversion. Beyond substrate-driven selection, host immune regulation may also participate in shaping these community patterns. Recent evidence suggests that immune–microbiota interactions can influence microbial composition under nutritional stress, contributing to the maintenance of ruminal homeostasis (62).

### 4.7 Associations linking microbes to fermentation

The two opposed clusters observed in the correlation map synthesize how diet directs microbial networks and fermentation outputs, thereby modulating the host's rumen environment and nutrition utilization. The propionate/NH_3_-N-linked cluster (*Ruminococcus*–*Prevotellaceae_UCG-001*–*Selenomonas*–*Rikenellaceae_RC9*) likely reflects a faster-turnover carbohydrate niche with active proteolysis and branched-chain amino acid catabolism, elevating isovalerate and valerate precursors (47). Such activity may enhance glucogenic energy supply and nitrogen recycling efficiency in the host. The acetate/butyrate/TVFA-linked cluster (*Bifidobacterium*–*Succiniclasticum*–*Methanobrevibacter*–*Dialister*) is more consistent with fiber fermentation and hydrogen-dependent cross-feeding where methanogens maintain low hydrogen partial pressures to sustain NADH reoxidation and butyrogenesis (63, 64). This pathway supports rumen pH stability and butyrate provision for epithelial integrity. The negative relationship between the two clusters underscores classical thermodynamic partitioning: propionogenesis competes with methanogenesis for reducing equivalents, whereas acetate/butyrate pathways are favored when hydrogen is efficiently removed, maintaining higher pH and total SCFA (45). Such shifts in electron flow directly influence fermentation efficiency and rumen homeostasis. Our data suggest that ST50 group nudged the system toward the former cluster—increasing propionate without lowering pH—whereas ST100 group trended toward the latter but with reduced TVFA, possibly reflecting insufficient fermentable nitrogen and residual lignification of the straw that limited rates. These microbial and metabolic changes may help maintain a balanced rumen environment and improve nutrient utilization in the ST50 group.

## 5 Conclusion

Replacing peanut vines with fermented rice straw modestly restructured the rumen microbiota and altered fermentation patterns without affecting overall diversity. A 5% inclusion improved glucogenic fermentation and maintained microbial stability, whereas 10% inclusion reduced digestibility, epithelial development, and feed efficiency. These results indicate that limited substitution is feasible for Hu lamb finishing diets, but higher levels may impair rumen function. Further optimization of straw pretreatment and nutrient synchronization is recommended to enhance its sustainable utilization.

## Data Availability

The names of the repository/repositories and accession number(s) can be found below: https://www.ncbi.nlm.nih.gov/, PRJNA1338370.
